# Teacher-Mediated Digital Game-Based Learning and Children’s Emotional Intelligence in Inclusive Classrooms: A Systematic Review and Critical Narrative Synthesis

**DOI:** 10.3390/jintelligence14080181

**Published:** 2026-08-07

**Authors:** Funda Uğurlu, Filiz Evran Acar

**Affiliations:** 1 School of Foreign Languages, Bandırma Onyedi Eylül University, 10200 Bandırma, Türkiye; 2 Department of Educational Sciences, Faculty of Education, Düzce University, 81620 Düzce, Türkiye; filizacar@duzce.edu.tr

**Keywords:** digital game-based learning, emotional intelligence, social–emotional learning, teacher mediation, inclusive education, serious games, PRISMA

## Abstract

Digital game-based learning is increasingly used to support children’s engagement, interaction, and social–emotional development. However, its contribution to emotional intelligence, particularly in inclusive classroom contexts, remains theoretically underdeveloped and methodologically fragmented. This systematic review and critical narrative synthesis examines how teacher- or facilitator-mediated digital games address children’s emotional intelligence and related social–emotional outcomes in inclusive educational contexts. Following PRISMA 2020 reporting guidance, the review examined peer-reviewed articles indexed in Web of Science and Scopus between 2015 and 2026. After screening 347 unique records and assessing 31 full-text reports, 14 studies were included in the final synthesis. The review focused on four interrelated areas: the emotional and social–emotional outcomes addressed in digital game-based studies; the roles of teachers or facilitators in structuring, mediating, and debriefing game-based activities; the inclusive classroom conditions that support equitable participation and emotional safety; and the risks, limitations, or implementation challenges associated with classroom use. The findings suggest that digital games may become educationally meaningful when they are linked to explicit emotional learning goals, embedded in structured classroom or school-based activities, supported by teacher or facilitator mediation, and followed by reflection or debriefing. Based on the synthesis, the review proposes a teacher-mediated inclusive digital game-based emotional learning framework consisting of seven dimensions: emotional learning target, game selection and pedagogical fit, teacher mediation, inclusive participation, classroom interaction, reflective debriefing, and risk monitoring.

## 1. Introduction

Emotional Intelligence (EI) and Social and Emotional Learning (SEL) are important for children’s holistic development, academic success, and psychological well-being (Cipriano et al., 2023; Durlak et al., 2011; Taylor et al., 2017). Theoretically, EI is defined as the capacity to perceive, understand, and regulate emotions, and to use emotional information to guide thought and behaviour (Mayer et al., 2004; Salovey & Mayer, 1990). In educational settings, SEL provides a broader practical framework for developing EI-related and other social–emotional competencies through five domains: self-awareness, self-management, social awareness, relationship skills, and responsible decision-making (Collaborative for Academic, Social, and Emotional Learning [CASEL], 2020; Durlak et al., 2025). In this review, emotional intelligence constitutes the primary theoretical lens, whereas SEL is treated as the broader educational context in which EI-related capacities may be developed and applied.

Cultivating the emotional abilities associated with emotional intelligence is particularly critical in inclusive classrooms, where educators must accommodate diverse cognitive and emotional profiles. For instance, students with diverse sensory, developmental, or attention-related needs may require different supports in terms of emotional regulation, executive functioning, accessibility, and peer interaction (Jiang et al., 2026; Manitsa et al., 2024; Meurer et al., 2026). For these learners, less flexible instructional approaches may fail to address differences in access, emotional regulation, and peer participation, thereby increasing the need for targeted, accessible, and engaging pedagogical alternatives (Jiang et al., 2026; Meyer et al., 2014; UNESCO, 2020).

Digital Game-Based Learning (DGBL) and serious games are increasingly used as pedagogical approaches that can respond to these challenges, offering immersive environments that foster cognitive, affective, and sociocultural engagement (Boyle et al., 2016; Clark et al., 2016; Plass et al., 2015; Wouters et al., 2013). Recent studies illustrate the potential of serious games tailored for inclusive educational settings. For example, accessible platforms may foster self-advocacy and social inclusion for visually impaired students in schools (Manitsa et al., 2024); trauma-informed game-based school programmes may support resilience among vulnerable youth (Meurer et al., 2026); and games designed for bullying prevention may promote perspective-taking and compassion (Paracha et al., 2023; Rončević Zubković et al., 2022). These environments can allow children to explore, make mistakes, try different responses, and practice emotion regulation and other self-regulatory skills within low-risk scenarios (Plass et al., 2015).

However, digital games should not be treated as self-sufficient learning tools (Beavis et al., 2014), since integrating technology into the classroom does not, by itself, guarantee positive emotional or social development. In fact, poorly implemented gamification—the use of specific game elements in non-game contexts—can yield mixed success and trigger adverse effects (Landers, 2014). Excessive reliance on extrinsic rewards, competitive leaderboards, and score visibility can induce anxiety, social comparison stress, and decreased intrinsic motivation (Hanus & Fox, 2015). Furthermore, studies suggest that specific gamification elements may not automatically foster intrinsic motivation or psychological need satisfaction, and their effects can depend on how they are designed and pedagogically framed (Mekler et al., 2017; Sailer et al., 2017). Taken together, these findings suggest that the efficacy of DGBL lies not merely in the medium itself, but in its instructional design, psychological foundations, and pedagogical integration (Clark et al., 2016; Landers, 2014).

Consequently, the pedagogical role of the teacher is indispensable. Research consistently shows that instructional support and teacher scaffolding are important conditions that enhance learning in game-based environments (Wouters & van Oostendorp, 2013). Furthermore, structured debriefing is critically important; as Crookall (2010) highlights, meaningful and lasting learning comes not just from the game itself, but from processing the game experience during post-game debriefing sessions. Teachers act as effective facilitators who create emotionally secure environments, guide students to focus on personal growth rather than peer comparison, and help them connect virtual dilemmas to real-life social interactions that promote emotional understanding and regulation through reflective discussions (Crookall, 2010; Hromek & Roffey, 2009).

Despite increasing empirical interest in digital games and serious games for emotional and social learning, their contribution to children’s emotional intelligence remains only partially theorized and methodologically fragmented, particularly in relation to how teachers mediate these digital tools within inclusive classrooms with diverse learner profiles. Following PRISMA 2020 reporting guidelines (Page et al., 2021), this review synthesizes peer-reviewed evidence indexed in Web of Science and Scopus and published between 2015 and 2026. It focuses on four interrelated issues: emotional intelligence and related emotional and social outcomes addressed in digital game-based studies; the pedagogical role of teachers or facilitators in structuring, mediating, and debriefing game-based activities; the inclusive classroom conditions that support equitable participation and emotional safety; and the risks, limitations, or implementation challenges that might restrict responsible use. By integrating these findings, this review proposes a teacher-mediated inclusive digital game-based emotional learning framework that identifies the pedagogical conditions under which digital games may foster social–emotional competencies grounded in emotional intelligence within inclusive classrooms.

## 2. Materials and Methods

### 2.1. Review Design

This study was designed as a systematic review with a critical narrative synthesis and was conducted in accordance with the PRISMA 2020 reporting guidelines. The review aimed to identify, screen, and synthesize peer-reviewed research on the relationship between teacher-mediated digital game-based learning and children’s emotional intelligence in inclusive classroom-relevant contexts. The PRISMA 2020 framework guided the identification, screening, eligibility assessment, inclusion, and reporting procedures (Page et al., 2021). This review was not prospectively registered, and no separate review protocol was published. Nevertheless, the search strategy, eligibility criteria, screening procedures, data extraction, and synthesis approach were established before study selection and are reported transparently in the Section 2 and Appendix A.

The review was structured to balance sensitivity and specificity. Initial exploratory searches produced a broad and heterogeneous body of literature, including studies on emotional intelligence, social–emotional learning, empathy, gamification, virtual reality, augmented reality, digital gaming, problematic gaming, and digital media-related risks. Therefore, the final search strategy was refined by combining three core conceptual blocks: emotion-related constructs, digital game-based learning constructs, and child/school-related educational contexts. This strategy was intended to reduce the retrieval of irrelevant records from adult, clinical, general technology, and non-educational gaming literature while retaining studies relevant to children’s emotional intelligence and emotional and social development in educational settings.

Terms related to teacher mediation and inclusive classroom conditions were not used as mandatory search blocks because these dimensions are often discussed in the full text rather than consistently indexed in titles, abstracts, or keywords. They were therefore applied during title–abstract screening, full-text eligibility assessment, data extraction, and narrative synthesis.

### 2.2. Data Sources and Search Strategy

The literature search was conducted in Web of Science and Scopus, which were selected because of their interdisciplinary coverage of peer-reviewed research in education, psychology, educational technology, computer science, social sciences, and child development. These databases were also selected because they provide structured metadata export formats compatible with BiBLoX-based bibliometric organization and screening.

**Figure 1:**
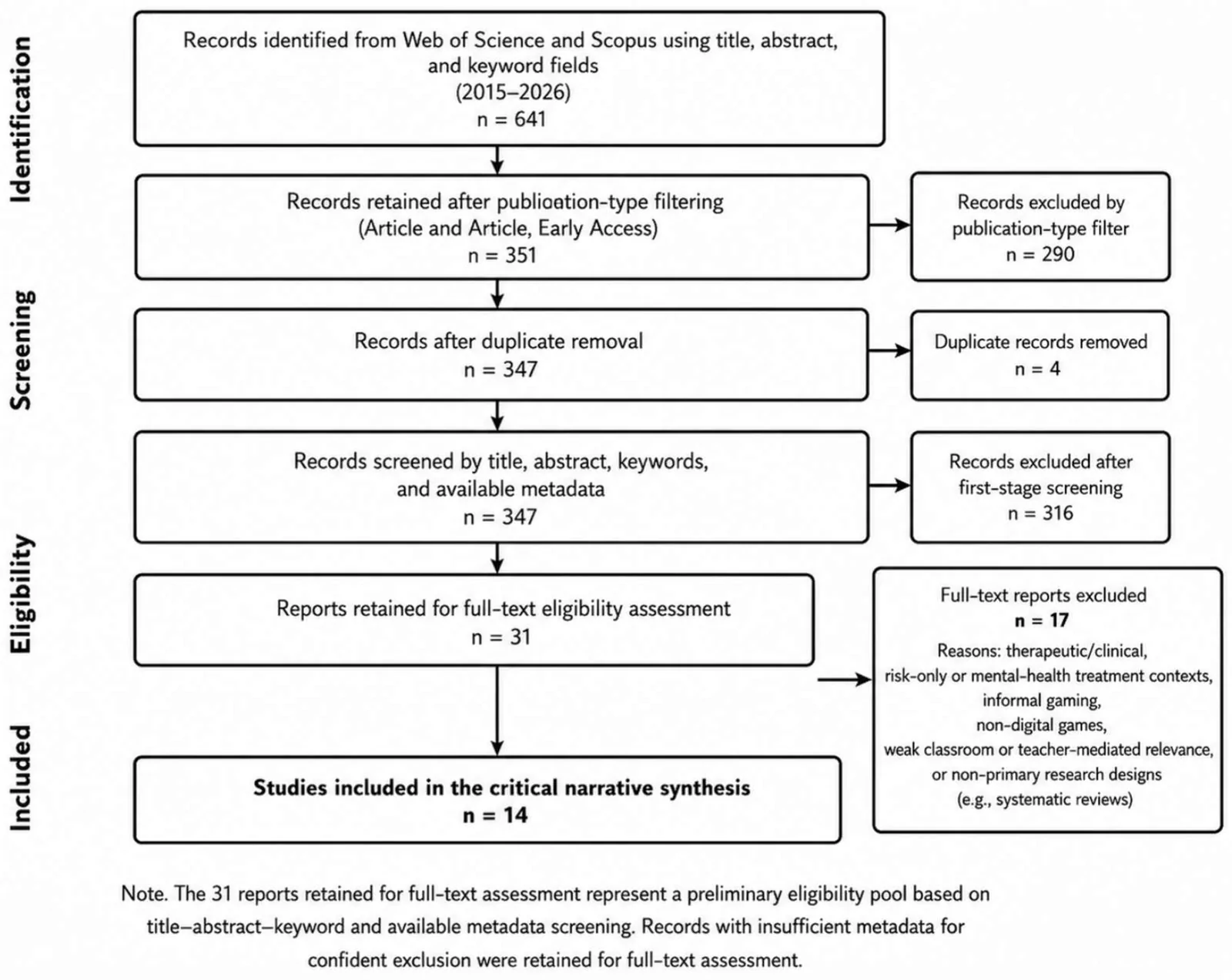
PRISMA 2020 flow diagram representing the record selection process.

The final search strategy was developed after preliminary testing of broader search strings. Earlier searches based on only two conceptual blocks—emotion-related constructs and digital game-related constructs—retrieved an excessively broad set of records, including many studies on adult gaming, clinical treatment, problematic gaming, digital addiction, and non-educational technology use. To improve precision, a third conceptual block relating to children, school students, and school-based educational contexts was added. The search strategy, therefore, combined three conceptual blocks: emotion-related constructs (including emotional intelligence and social–emotional learning), digital game-based learning constructs, and child/school-related educational contexts. The full database-specific search strings used in Web of Science and Scopus are provided in Appendix A, Table A1. Publication year, language, and document-type restrictions were applied through database filters rather than embedded in the search strings. The complete retrieval, deduplication, screening, eligibility, and inclusion counts are reported in the PRISMA flow diagram in Figure 1.

### 2.3. BiBLoX-Based Data Organization

The retrieved records were organized and processed using BiBLoX, a bibliometric analysis and literature organization platform designed to support automated bibliometric analysis, trend detection, metadata cleaning, duplicate checking, and dataset segmentation. In line with the use of BiBLoX in previous bibliometric work, the platform was used in this study to import metadata from Web of Science and Scopus, organize publication records, examine descriptive trends, and prepare the dataset for PRISMA-based screening (Kesgin & Ozer, 2025).

BiBLoX was used to generate preliminary descriptive indicators, including annual publication trends, citation patterns, publication outlet distributions, keyword frequencies, and author-level outputs. These analyses were not treated as evidence of intervention effectiveness. Instead, they were used to describe the broader structure of the retrieved literature and to support transparent organization of the review corpus before manual eligibility assessment. Because BiBLoX-assisted classification and visualization rely on bibliographic metadata and abstract-level information, all substantive inclusion decisions were made through manual title–abstract screening and full-text eligibility assessment. Thus, BiBLoX supported the organization and descriptive profiling of the dataset, whereas final inclusion decisions were based on the predefined eligibility criteria.

### 2.4. Inclusion and Exclusion Criteria

The inclusion and exclusion criteria were established before the screening process and were applied in two stages. First, the studies in the BiBLoX-exported working dataset were screened on the basis of titles, abstracts, and keywords. Second, potentially relevant studies were assessed through full-text eligibility review to confirm population characteristics, intervention type, emotional or social–emotional outcomes, educational context, and methodological sufficiency. This two-stage procedure ensured that the broad search strategy was followed by a transparent and consistent selection process. Table 1 summarizes the operationalized inclusion and exclusion criteria used during screening and eligibility assessment.

The criteria in Table 1 were applied first during title–abstract–keyword screening and subsequently during full-text eligibility assessment. The screening procedure is described in Section 2.6.

### 2.5. Publication Types

The review included peer-reviewed journal articles and early access articles indexed in Web of Science and Scopus. The decision to restrict the final corpus to articles and early access articles was made to improve methodological comparability and ensure that included studies provided sufficient information on research design, participants, intervention or learning activity, outcome measures, and findings.

Conference papers, reviews, review articles in early access, book chapters, books, conference reviews, meeting abstracts, editorials, editorial materials, notes, letters, abstracts, and other non-empirical or non-article publication types were excluded from the final synthesis to maintain consistency in publication type and reduce variability in reporting depth. Relevant review articles were not included as primary evidence but were consulted where appropriate for background contextualization and reference tracking.

### 2.6. Screening Procedure and PRISMA Flow

The screening process followed PRISMA 2020 reporting principles. One reviewer screened the records and assessed potentially eligible full texts, while a second reviewer checked the eligibility decisions. Uncertain cases and disagreements were resolved through discussion and consensus. BiBLoX was used to organize the records, support duplicate identification, and prepare the working dataset; it was not used to make automated inclusion or exclusion decisions.

After publication-type filtering, duplicate records were removed and the remaining records were screened manually by title, abstract, keywords, and available metadata against the predefined eligibility criteria presented in Table 1. Records were excluded when they clearly lacked a relevant digital game-based educational component, an emotion-related outcome, a school-age population, or an educationally relevant context. Records with incomplete metadata were retained when the available information was insufficient for confident exclusion.

The full texts of the remaining reports were then assessed for eligibility. This assessment considered the population, digital game-based component, emotion-related focus, educational relevance, teacher or facilitator role, and adequacy of methodological reporting. Reports that did not meet these criteria were excluded, and the remaining studies were included in the critical narrative synthesis. The complete study selection process is presented in the PRISMA 2020 flow diagram (Figure 1).

### 2.7. Data Extraction

A structured study-level data extraction form was applied to all articles retained after full-text eligibility assessment to capture the demographic, methodological, intervention, and outcome information required for the critical narrative synthesis. Data extraction from each included report was conducted collaboratively and in a coordinated manner by two researchers using the same structured extraction form; the researchers did not work independently. Extracted information included the research context, educational setting, participant characteristics, study design, sampling, intervention characteristics, comparison condition where applicable, implementation details, teacher or facilitator role, data-collection methods, outcome measures, findings, attrition, and author-reported limitations. Review-specific information was also extracted, including the type of digital game or game-based learning activity, intervention duration, emotion-related outcomes—including emotional intelligence and related social–emotional outcomes where reported—measurement instruments, and key findings.

Outcome extraction preserved the reported constructs, measurement instruments, directions of findings, and supporting quantitative or qualitative evidence. Quantitative data included effect estimates, test statistics, confidence intervals, significance levels, or descriptive changes where available. Qualitative and design-oriented studies contributed themes, usability findings, stakeholder recommendations, accessibility requirements, design outputs, and implementation findings. Intended objectives were not treated as achieved outcomes unless supported by reported evidence. Missing information was recorded as not reported (NR), and participant or study characteristics were not inferred from contextual information. Where multiple publications appeared to report findings from related samples, they were examined carefully to avoid treating the same evidence as independent studies.

### 2.8. Data Synthesis

Given the conceptual and methodological diversity of the studies retained after full-text eligibility assessment, a critical narrative synthesis was conducted rather than a statistical meta-analysis. Meta-analysis was considered inappropriate because the included studies differed substantially in participant age groups, educational levels, intervention types, digital game formats, learning contexts, outcome constructs, measurement instruments, and research designs. The synthesis therefore focused on identifying patterns, contrasts, and pedagogical conditions across studies rather than estimating a pooled effect size. Because the studies differed in their capacity to support causal, comparative, descriptive, design, or implementation inferences, they were not treated as methodologically equivalent. Recurring patterns across different contexts and designs were interpreted as potentially meaningful but not as proof of effectiveness.

The synthesis was organized around the review questions and proceeded through iterative comparison of study aims, participant characteristics, intervention features, educational contexts, teacher or facilitator roles, emotion-related outcomes—including emotional intelligence and related social–emotional outcomes where reported—findings, and study limitations. Particular attention was given to how digital games were positioned within each study: as stand-alone technological tools, structured educational activities, assessment environments, or pedagogically mediated learning experiences.

All descriptive and analytic coding was conducted collaboratively by two subject-matter experts, who reviewed and agreed on the final codes and their consolidation into the framework dimensions. Descriptive coding captured each study’s research context, educational level, participant characteristics, design, game-based intervention, implementation setting, duration, teacher or facilitator role, outcome measures, findings, and limitations. Analytic coding focused on recurring pedagogical, emotional, and inclusion-related features across the included studies. These included teacher mediation, inclusive participation, classroom interaction, emotional safety, peer collaboration, competition, score visibility, social comparison, frustration, exclusion, distraction, and problematic game engagement. Codes were assigned only when supported by the study design, implementation, findings, or reported limitations; an absence of reporting was not interpreted as evidence that a feature was absent.

The studies were subsequently grouped according to recurring thematic dimensions, including digital game-based approaches, targeted emotion-related outcomes, teacher mediation and pedagogical structuring, inclusive participation and classroom conditions, and reported risks, constraints, or implementation challenges. This process enabled the synthesis to examine not only whether digital games were associated with emotion-related learning but also the pedagogical and contextual conditions under which such learning was supported or constrained. The final analytic codes, their consolidation into the dimensions of the proposed framework, and the studies contributing to each dimension are presented in detail in Section 3.8.

In the final stage, the thematic findings were integrated to develop a teacher-mediated, inclusive digital game-based emotional learning framework. The framework was derived from recurring patterns across the included studies and was used to organize the interpretation of how digital games can be responsibly integrated into classroom or school-based contexts to support children’s emotional intelligence while minimizing risks related to exclusion, frustration, excessive competition, distraction, social comparison, emotional discomfort, and problematic engagement. The resulting framework should therefore be interpreted as an organizational structure derived from the critical narrative synthesis rather than as a statistically or psychometrically validated model. A formal risk-of-bias tool was not applied; instead, the design-sensitive methodological appraisal described in Section 2.9 was used to inform the interpretation of the evidence.

### 2.9. Methodological Quality Appraisal

This procedure constituted a descriptive methodological appraisal rather than a formal study-level risk-of-bias assessment. Because the included studies employed heterogeneous designs—including intervention, assessment, participatory and co-design, intervention-development, usability, feasibility, curriculum, and mixed-methods studies—a single risk-of-bias instrument was not applied across the corpus, as this would have produced non-equivalent and potentially misleading judgements. Instead, a design-sensitive descriptive appraisal evaluated each study across six domains: the appropriateness of the research design; the reporting of sample and participant characteristics; the presence of a comparison group, randomization, or baseline assessment where applicable; the quality and transparency of outcome measures and data-collection procedures; the clarity of intervention, implementation, facilitator, and analytical reporting; and acknowledged limitations, including selection bias, confounding, attrition, short follow-up, self-report bias, limited generalizability, and the absence of effectiveness testing. The appraisal was interpretive rather than exclusionary and did not generate numerical quality scores because the relevance of individual domains varied by study design. Accordingly, findings were interpreted according to the type of evidence each design could support: comparative intervention studies contributed primarily to the evaluation of possible outcome changes, whereas design, co-design, assessment, feasibility, and implementation studies informed pedagogical fit, acceptability, accessibility, teacher mediation, and implementation conditions. The appraisal results are summarized in Section Methodological Quality and Limitations of the Included Evidence and Table 2.

## 3. Results

### 3.1. Study Selection and Eligibility Outcomes

The initial searches of Web of Science and Scopus, conducted within the 2015–2026 publication period using title, abstract, and keyword fields, identified 641 records. After the publication-type filter was applied to retain only journal articles and early access articles, 351 records remained and were exported to the working dataset. Following the removal of four duplicates, 347 unique records were available for title–abstract screening.

During this first-stage screening, 316 records were excluded because they did not meet the predefined eligibility criteria. The principal reasons were the absence of a digital game-based or gamified educational component, the absence of emotional intelligence or related social–emotional learning outcomes, adult or university-level samples, non-school or informal gaming contexts, and a risk-only or pathology-oriented focus without clear pedagogical relevance.

The remaining 31 reports were retained for full-text eligibility assessment. These reports constituted a preliminary eligibility pool because records with incomplete abstract metadata were retained when the available information did not permit confident exclusion during title–abstract screening. Following full-text assessment, 17 reports were excluded because they concerned therapeutic or clinical interventions, risk-only or mental-health treatment contexts, informal gaming, non-digital games, weak classroom or teacher-mediated relevance, or non-primary research designs such as systematic reviews. These excluded studies are presented in Supplementary Table S1 (Álvarez Muñoz et al., 2025; Beaumont et al., 2021; Black et al., 2025; Bohr et al., 2023; Çar et al., 2023; David et al., 2018, 2024; Gómez León, 2024; Grossard et al., 2017a, 2017b; Grunewald et al., 2025; Hassan et al., 2025; Hernandez-Lara et al., 2023; Livanou et al., 2025; Muravevskaia & Gardner-McCune, 2023; Nagpal et al., 2026; Novianto et al., 2026). Finally, fourteen studies met the final inclusion criteria and were included in the critical narrative synthesis. The included studies represented classroom-relevant, school-based, or pedagogically structured uses of digital games, serious games, gamified emotional education software, game-based assessment, or game-based curricula to support emotional intelligence, empathy, emotion regulation, social skills, peer interaction, inclusion, resilience, or related social–emotional outcomes. These included studies provided the evidence base for the subsequent thematic synthesis (Chao-Fernández et al., 2020; Craig et al., 2015; Filella Guiu et al., 2016; Ghanat et al., 2025; Manitsa et al., 2024; Meurer et al., 2026; Paracha et al., 2019, 2020, 2023; Rihlah et al., 2026; Rončević Zubković et al., 2022; Ros-Morente et al., 2018; Saleme et al., 2020, 2021). The study selection process is summarized in Figure 1.

### 3.2. General Characteristics of the Included Studies

The critical narrative synthesis included 14 primary studies published between 2015 and 2026. The studies covered a range of countries and educational contexts and involved learners from early childhood through secondary education, including both general school populations and learners with sensory or other support needs. Reporting of participant characteristics was inconsistent: some studies provided detailed information on sample size, age, and sex, whereas others reported only school grade, target age, or participant numbers for specific study phases. This uneven reporting limited systematic comparisons by sex, age, and learner group. Table 2 presents the research context, participant characteristics, design, intervention, outcome measures, principal findings, and evidential contribution of each included study.

The included studies used a broad range of research designs, including comparative and non-comparative interventions, pre–post evaluations, game-based assessments, participatory and co-design studies, intervention-development research, usability and feasibility evaluations, curriculum pilots, and a mixed-methods school programme. Participant numbers reflected different forms of involvement across these designs. In intervention studies, they generally represented learners included in outcome evaluations, whereas in design and development studies they referred to children or stakeholders involved in design, usability, accessibility, or implementation activities.

The interventions included emotional-education software, serious games, virtual environments, computer game-supported activities, game-based assessments, inclusive educational games, game-based curricula, and multicomponent school programmes. Their duration ranged from single-session activities to extended interventions, and the involvement of teachers, facilitators, researchers, and programme staff varied considerably. The studies addressed emotion recognition, emotion regulation, emotional competence, empathy, social skills, prosocial behaviour, resilience, well-being, character development, and social inclusion through diverse instruments and procedures. The methodological quality, evidential contribution, and limitations associated with these different study designs are examined separately in Section Methodological Quality and Limitations of the Included Evidence.

#### Methodological Quality and Limitations of the Included Evidence

The design-sensitive appraisal indicated substantial variation in the methodological quality and evidential contribution of the included studies. Comparative and repeated-measures studies provided a relatively stronger basis for considering possible changes in emotional or social outcomes. Filella Guiu et al. (2016), Ros-Morente et al. (2018), Rončević Zubković et al. (2022), Saleme et al. (2020), Ghanat et al. (2025), Rihlah et al. (2026), and Meurer et al. (2026) used pre–post assessments, comparison conditions, structured programme evaluations, or mixed methods. Their strengths included school-based implementation, clearly specified outcomes, comparison or baseline data, and, in some cases, relatively large samples or multiple sources of evidence. However, several were non-randomized, uncontrolled, short-term, or based on small and context-specific samples. Their findings were therefore vulnerable to selection effects, baseline differences, implementation variation, self-report bias, limited follow-up, and restricted generalizability. In multicomponent programmes such as Meurer et al. (2026), the independent contribution of the game could not be separated from mentoring and other programme elements. Saleme et al. (2020) also provided only short-term evidence from a single-session, uncontrolled pilot.

Other studies contributed primarily to design, development, usability, feasibility, accessibility, or implementation rather than intervention effectiveness. Paracha et al. (2019, 2020, 2023), Saleme et al. (2021), and Manitsa et al. (2024) involved participatory design, theory-informed intervention development, prototype evaluation, stakeholder involvement, or child-centred usability work. These approaches provided relevant evidence concerning children’s agency, pedagogical design, acceptability, emotional safety, and accessibility. Nevertheless, their formative and context-specific designs did not establish sustained improvements in emotional or social competencies.

Craig et al. (2015) made a different methodological contribution by using a standardized game-based assessment to examine social-skills performance across cultural groups. Its relatively large sample, direct behavioural tasks, and cross-cultural comparison strengthened the assessment evidence. However, because the study evaluated performance rather than an intervention, its findings did not demonstrate that gameplay improved social skills. Chao-Fernández et al. (2020) provided classroom-based pre–post evidence concerning emotional and behavioural outcomes, but its very small, uncontrolled sample limited causal inference and long-term generalizability.

Across the corpus, recurring limitations included convenience recruitment, incomplete reporting of participant characteristics, heterogeneous outcome definitions, reliance on self-report or study-specific measures, and limited reporting of attrition, implementation fidelity, teacher mediation, reflective debriefing, accessibility procedures, and adverse effects. Independent replication was also limited. Variation in the reported findings was considered in relation to differences in study design, participant characteristics, educational setting, game format, intervention duration, outcome definitions, measurement instruments, and the extent of teacher or facilitator involvement. Because these factors frequently overlapped across a small and heterogeneous evidence base, no single source of variation could be identified as explaining the differences among study findings.

Consequently, the appraisal provides greater confidence in the identification of recurring pedagogical, accessibility, design, and implementation considerations than in broad claims of intervention effectiveness. The evidence identifies promising practices and relevant implementation boundaries but does not support a general causal conclusion that digital games consistently improve children’s emotional and social competencies.

### 3.3. Digital Game-Based Approaches for Supporting Emotional Intelligence and Related Emotional and Social Learning

The included studies showed that digital game-based approaches targeted emotional intelligence and related emotional and social learning outcomes through diverse technological and pedagogical formats rather than through a single type of digital game. The approaches identified in the final synthesis can therefore be grouped into seven descriptive categories reflecting how the games were designed and used: gamified emotional education software, serious games and virtual environments for empathy and peer-related learning, participatory design and co-design for SEL, game-based assessment tools, computer- or video-game-based emotional training, inclusive educational games for diverse learners, and school-based game curricula or broader programmes. These categories describe the main forms of game-based activity represented in the included studies.

Some studies used gamified emotional education software to support emotional competence, emotional regulation, well-being, and peer conflict resolution. For example, Happy 8–12 was used as an emotional education programme to support assertive conflict resolution among peers, while other gamified emotional education applications addressed emotional and well-being variables among children and adolescents (Filella Guiu et al., 2016; Ros-Morente et al., 2018).

A second group of studies used serious games, virtual environments, or gamified activities to address empathy, bullying prevention, moral reasoning, compassion, prosocial behaviour, and emotional safety. Although these outcomes are commonly discussed within social and emotional learning (SEL) frameworks, they were retained in this review because they represent emotion-related competencies examined in the included studies. These studies positioned digital games as scenario-based environments in which children could encounter social dilemmas, interpret peer perspectives, and reflect on the emotional consequences of actions. This pattern was visible in studies focusing on empathy promotion, socio-emotional skills, bullying prevention, and moral, social, and emotional learning (Paracha et al., 2020, 2023; Rončević Zubković et al., 2022; Saleme et al., 2020, 2021).

A distinct approach involved participatory design and co-design for SEL. Paracha et al. (2019) used child-centred participatory design to connect design thinking with social and emotional learning. This approach is important because it positions children not only as users of game-based learning environments but also as active contributors to the design and meaning-making process.

A smaller but important strand of the literature used digital games for assessment or observation of social–emotional skills. Craig et al. (2015), for instance, used a game-based social skills assessment to examine children’s social competence through structured game tasks. This assessment-oriented use of game environments was retained in the synthesis because it shows how digital games can make social behaviours and emotional competencies observable in ways that are difficult to capture through traditional self-report measures alone.

Another group of studies focused on computer- or video-game-based emotional training. Chao-Fernández et al. (2020) used a computer-game-based music activity to support emotional training and reduce disruptive behaviours in secondary education. This study shows that digital games can be integrated into classroom activities not only for direct SEL instruction but also for emotional engagement and behaviour-related support.

Inclusive or accessibility-oriented applications formed another category. These studies examined how educational games or computer games could support children with diverse sensory, developmental, or participation needs. Manitsa et al. (2024) examined an educational game designed to support the social inclusion of students with vision impairment, while Ghanat et al. (2025) focused on a computer game for recognizing and understanding facial emotions among hearing-impaired children. These studies were particularly relevant to the review’s focus on inclusive participation and differentiated access to emotional and social learning.

Finally, several studies examined school-based curricula or broader educational programmes that integrated game-based learning into structured pedagogical contexts. Meurer et al. (2026) examined a trauma-informed school programme integrating mentorship and game-based learning to foster resilience and emotional intelligence. Rihlah et al. (2026) also contributed to this category through an AI-based edugame designed for early childhood education and character development. These studies suggest that digital games may be most educationally meaningful when embedded within broader instructional, developmental, or school-based support structures.

As a result, the included studies illustrate substantial variation in the pedagogical structuring, teacher or facilitator involvement, technological format, and the emotion-related outcomes they sought to support. This variation shows the breadth of possible applications but prevents direct comparison of the intervention categories or identification of a generally superior game format. The findings should therefore be interpreted as a descriptive mapping of approaches represented in the literature rather than as evidence that each approach is effective or that similar outcomes can be expected across populations and settings.

### 3.4. Emotional Intelligence and Social–Emotional Outcomes

Whereas Section 3.3 classified the studies according to the format and pedagogical use of the digital game-based approach, this section organizes the specific emotional and social–emotional outcomes examined or reported across the included studies. The included studies addressed a range of emotional intelligence and social–emotional learning outcomes. These outcomes clustered around five main areas: emotional competence and emotion regulation, empathy and compassion, social skills and peer interaction, prosocial behaviour and moral reasoning, and resilience or emotional well-being.

Emotional competence and emotion regulation were among the most frequently addressed outcomes. Gamified emotional education software was used to support children’s emotional awareness, emotional understanding, and peer conflict resolution (Filella Guiu et al., 2016; Ros-Morente et al., 2018). Trauma-informed game-based learning was linked to resilience, emotional intelligence, and emotion regulation in school contexts (Meurer et al., 2026).

Empathy and compassion formed a second major outcome area. Several studies used serious games, gamified approaches, or virtual environments to promote empathy, perspective-taking, bullying awareness, and compassion among children (Paracha et al., 2020, 2023; Rončević Zubković et al., 2022; Saleme et al., 2020, 2021). These studies suggest that digital games can create structured social scenarios in which learners encounter emotional dilemmas and reflect on the consequences of their responses.

Social skills, peer interaction, and inclusion were also prominent. Craig et al. (2015) used a game-based assessment to examine children’s social skills, while Manitsa et al. (2024) focused on social inclusion at school for students with vision impairment. Ghanat et al. (2025) extended this inclusive focus by examining emotion recognition and understanding among hearing-impaired children through a computer game.

Finally, some studies addressed broader developmental outcomes such as social–emotional well-being, character development, and prosocial behaviour. These were particularly visible in game-based SEL curricula, AI-based edugames, and school-based programmes that embedded digital games within wider educational or developmental structures (Rihlah et al., 2026; Saleme et al., 2021).

In essence, the synthesized evidence indicates that digital game-based approaches were primarily designed to support specific emotion-related competencies rather than emotional intelligence as a single global construct. Across the included studies, the most frequently targeted outcomes were emotional competence, emotion regulation, empathy, social skills, peer interaction, resilience, and prosocial behaviour. Although these outcomes were conceptualized differently across studies, they consistently reflected the application of digital games to support children’s emotional functioning within educational contexts.

### 3.5. Teacher Mediation and Pedagogical Structuring

The included studies indicate that digital games supported emotional and social learning most clearly when they were embedded in a pedagogically structured process rather than used as stand-alone digital tools. Teacher or facilitator mediation appeared in different forms, including preparing learners for the activity, organizing participation, guiding reflection, connecting the game experience to emotional or social concepts, and creating a safe environment for discussion.

In several studies, pedagogical mediation was visible through the design of structured emotional education or structured SEL activities. For example, gamified emotional education software was used to support peer conflict resolution and emotional competence when embedded in an educational programme rather than presented as isolated play (Filella Guiu et al., 2016; Ros-Morente et al., 2018). Similarly, school-based or curriculum-based studies showed that digital games became more meaningful when integrated into broader instructional or developmental frameworks (Meurer et al., 2026).

Facilitator or educator guidance was also important in studies focusing on empathy, bullying prevention, and prosocial behaviour. These studies often used scenarios, reflection, or guided interaction to help students interpret emotions, consider others’ perspectives, and connect game experiences to social consequences (Paracha et al., 2019, 2020, 2023; Rončević Zubković et al., 2022; Saleme et al., 2020, 2021). In this sense, the teacher or facilitator role was not limited to technical supervision; it involved transforming game-based experiences into opportunities for emotional interpretation and social learning.

In inclusive education contexts, mediation was particularly important because students’ access, participation, and emotional safety depended on how the activity was structured. Studies involving students with vision impairment or hearing impairment showed that digital games can support inclusion and emotion-related learning when accessibility, guidance, and differentiated participation are considered (Ghanat et al., 2025; Manitsa et al., 2024).

Collectively, the findings suggest that teacher or facilitator mediation may be an important condition for transforming digital gameplay into emotional and social learning. However, mediation was not examined as an independent variable in most studies.

Across several included studies, pedagogically structured implementation involved explicit learning goals, guided participation, interpretation of game experiences, and reflective or developmental follow-up.

### 3.6. Inclusive Participation and Classroom Conditions

The included studies indicate that digital games can create opportunities for participation, peer interaction, and emotional expression when they are accessible, pedagogically structured, and connected to classroom or school-based learning goals. However, inclusion was not an automatic consequence of digital game use; rather, it depended on how games were designed, facilitated, and adapted to learners’ needs.

Several studies focused directly on learners with diverse needs. Manitsa et al. (2024) examined an educational game designed to support the social inclusion of students with vision impairment, while Ghanat et al. (2025) used a computer game to support facial emotion recognition and understanding among hearing-impaired children. These studies show that digital games may support inclusive emotional learning when accessibility and differentiated participation are explicitly considered.

Inclusive classroom conditions were also evident in studies addressing peer interaction, bullying prevention, empathy, and social skills. Serious games and gamified activities created structured situations in which children could consider others’ feelings, reflect on social consequences, and engage with peer-related dilemmas (Paracha et al., 2020, 2023; Rončević Zubković et al., 2022; Saleme et al., 2020, 2021). Similarly, game-based social skills assessment and emotional education software offered structured ways to observe or support children’s social participation and peer conflict resolution (Craig et al., 2015; Filella Guiu et al., 2016).

The classroom or school context was especially important in studies where game-based learning was embedded in broader SEL, resilience, or character-development programmes. In these studies, inclusive participation was supported not only through access to digital games but also through emotional safety, teacher or facilitator guidance, and opportunities for discussion and reflection (Meurer et al., 2026; Rihlah et al., 2026).

Overall, the findings indicate that inclusive digital game-based learning requires more than access to digital technologies. Across the included studies, inclusive participation was most consistently associated with accessible game design, structured participation, peer interaction, emotional safety, and teacher or facilitator guidance. Together, these conditions positioned digital games as inclusive learning environments rather than as stand-alone recreational technologies.

### 3.7. Reported Risks, Constraints, and Implementation Challenges

The included studies did not present digital game-based learning as uniformly beneficial. Rather, they consistently highlighted that reported outcomes were influenced by pedagogical design, teacher or facilitator mediation, accessibility, and attention to learners’ emotional safety. Although few studies directly examined adverse effects, several described implementation constraints related to accessibility, emotional sensitivity, classroom integration, and the transferability of findings.

One important challenge concerned the risk of superficial emotional learning when digital games were used without sufficient reflection or pedagogical follow-up. Studies focusing on empathy, prosocial behaviour, bullying prevention, and moral/social learning showed that game scenarios can support emotional understanding, but their educational value depends on how learners are guided to interpret social situations and connect in-game experiences to real-life peer relations (Paracha et al., 2020, 2023; Rončević Zubković et al., 2022; Saleme et al., 2020, 2021). Across these studies, guided discussion and pedagogical follow-up were presented as important components for linking in-game experiences with broader emotional and social learning objectives.

A second constraint involved accessibility and differentiated participation. Studies involving students with vision impairment or hearing impairment demonstrated the potential of digital games for inclusive participation, but they also highlighted the need for design adaptations, accessible interfaces, and adult support (Ghanat et al., 2025; Manitsa et al., 2024). These findings suggest that digital games may unintentionally exclude learners if accessibility, usability, and participation differences are not addressed during design and classroom implementation.

A third challenge related to emotionally sensitive content. Games addressing bullying, peer conflict, suicide prevention, trauma, or emotional regulation require careful facilitation because these topics may evoke discomfort, vulnerability, or strong emotional reactions among students (Filella Guiu et al., 2016; Meurer et al., 2026; Paracha et al., 2023; Rončević Zubković et al., 2022). In such cases, teacher or facilitator mediation is essential for maintaining emotional safety and preventing the activity from becoming distressing or stigmatizing.

Finally, several studies were limited by issues of generalizability and sustainability. Some interventions were design-oriented, pilot-based, or context-specific, while others focused on particular learner groups or specific school programmes (Craig et al., 2015; Rihlah et al., 2026; Ros-Morente et al., 2018). Collectively, these studies were characterized by context-specific designs, pilot implementations, or particular learner populations, which limited the transferability of their findings across educational settings.

In a nutshell, the synthesized evidence indicates that the reported constraints were primarily associated with implementation conditions rather than with digital game technologies themselves. Across the included studies, pedagogical mediation, accessibility, emotional safety, and integration within classroom or school-based practices emerged as the most frequently reported conditions influencing the implementation of digital game-based emotional and social learning.

### 3.8. Toward a Teacher-Mediated Inclusive Digital Game-Based Emotional Learning Framework

The synthesis of the 14 included studies suggests that digital games may support children’s emotional intelligence and social–emotional learning when embedded within a structured pedagogical process. Using the data-extraction and coding procedures described in Section 2.7 and Section 2.8, study-level analytic codes relating to emotional outcomes, game characteristics, pedagogical guidance, accessibility, peer interaction, reflection, and implementation constraints were consolidated into seven interrelated framework dimensions. Across the included studies, digital games appeared more educationally meaningful when they were connected to explicit emotional or social learning goals, supported by teacher or facilitator mediation, designed to enable inclusive participation, and accompanied by reflection or debriefing (Filella Guiu et al., 2016; Meurer et al., 2026; Ros-Morente et al., 2018).

Based on this synthesis, the review proposes a Teacher-Mediated Inclusive Digital Game-Based Emotional Learning Framework. The framework does not position digital games as inherently beneficial or harmful. Instead, it conceptualizes them as pedagogical environments whose educational value depends on how they are selected, structured, mediated, and evaluated within classroom or school contexts. The framework comprises seven interrelated dimensions: emotional learning target, game selection and pedagogical fit, teacher mediation, inclusive participation, classroom interaction, reflective debriefing, and risk monitoring.

Table 3 presents the audit trail linking the final analytic codes to the dimensions of the proposed framework, explains the logic through which the codes were consolidated, and identifies the included studies contributing to each dimension. The framework represents an interpretive synthesis rather than a validated measurement model; its dimensions are neither mutually exclusive nor addressed by every included study. Because formal debriefing and explicit risk-monitoring procedures were reported inconsistently, the relevant dimensions also encompass related evidence concerning facilitated reflection, emotional safety, accessibility, and implementation constraints.

Accordingly, the framework integrates recurring pedagogical considerations associated with emotional intelligence and related social–emotional learning outcomes across the reviewed evidence. It is, however, limited by the number and heterogeneity of the studies included. The seven dimensions interrelate to form theoretically consistent considerations derived from the synthesis, but their interaction, sequencing and relative importance have not been empirically validated. It should therefore be viewed as a framework for the development of hypotheses for future research and instructional design rather than as a validated intervention model.

The emotional learning target dimension concerns the intended emotion-related competencies and learning outcomes of the activity. The included studies targeted emotional competence, emotion regulation, empathy, compassion, peer interaction, prosocial behaviour, resilience, and social inclusion (Craig et al., 2015; Ghanat et al., 2025; Rončević Zubković et al., 2022; Saleme et al., 2021). The synthesis indicates the importance of aligning digital games with a clearly defined emotional or social–emotional learning purpose rather than using them solely to increase motivation or engagement.

Game selection and pedagogical fit concern the alignment of the game with the intended learning goals, learner characteristics, and developmental and educational context. For example, empathy and bullying-prevention games may be appropriate for perspective-taking and compassion, whereas emotional education software may be more suitable for emotion recognition, regulation, or conflict resolution (Filella Guiu et al., 2016; Paracha et al., 2023; Ros-Morente et al., 2018; Saleme et al., 2020). Accordingly, the framework proposes that game selection takes account of learners’ age, developmental needs, characteristics, and classroom context.

Teacher mediation concerns the preparation and organization of gameplay, guided participation, and instructional follow-up. The findings suggest that emotional learning may be strengthened when teachers or facilitators prepare learners, organize participation, guide in-game or post-game discussion, and help students connect game experiences to real-life emotions and peer relationships (Chao-Fernández et al., 2020; Meurer et al., 2026; Paracha et al., 2020). Without such mediation, digital games may remain engaging activities without producing deeper emotional understanding.

Inclusive participation emphasizes accessibility, differentiated engagement, and emotional safety. Studies involving students with vision impairment or hearing impairment showed that digital games can support inclusive emotional learning when accessibility and participation barriers are addressed (Ghanat et al., 2025; Manitsa et al., 2024). This dimension is particularly important because the same game may support some learners while excluding others if participation conditions are not carefully planned.

Classroom interaction refers to the social dynamics created during game-based activities. Many included studies used digital games to support peer interaction, prosocial behaviour, bullying awareness, compassion, and social inclusion (Paracha et al., 2019, 2023; Rončević Zubković et al., 2022; Saleme et al., 2021). This indicates that the classroom use of digital games should be evaluated not only by individual learning outcomes but also by how games shape peer relations and classroom climate.

Reflective debriefing concerns how learners interpret the emotional and social meaning of gameplay. Because formal debriefing was not consistently reported, this dimension also includes related practices such as guided discussion, facilitated interpretation, mentorship, reflection on decisions and consequences, and connecting game experiences to real-life relationships. These practices were particularly evident in studies addressing peer conflict, empathy, moral and social reasoning, bullying, compassion, and trauma-informed learning (Filella Guiu et al., 2016; Meurer et al., 2026; Paracha et al., 2019, 2020, 2023; Rončević Zubković et al., 2022; Saleme et al., 2020, 2021). Although reflective processing emerged as an important pedagogical condition, the evidence does not indicate that all interventions implemented a formal debriefing protocol.

**Figure 2:**
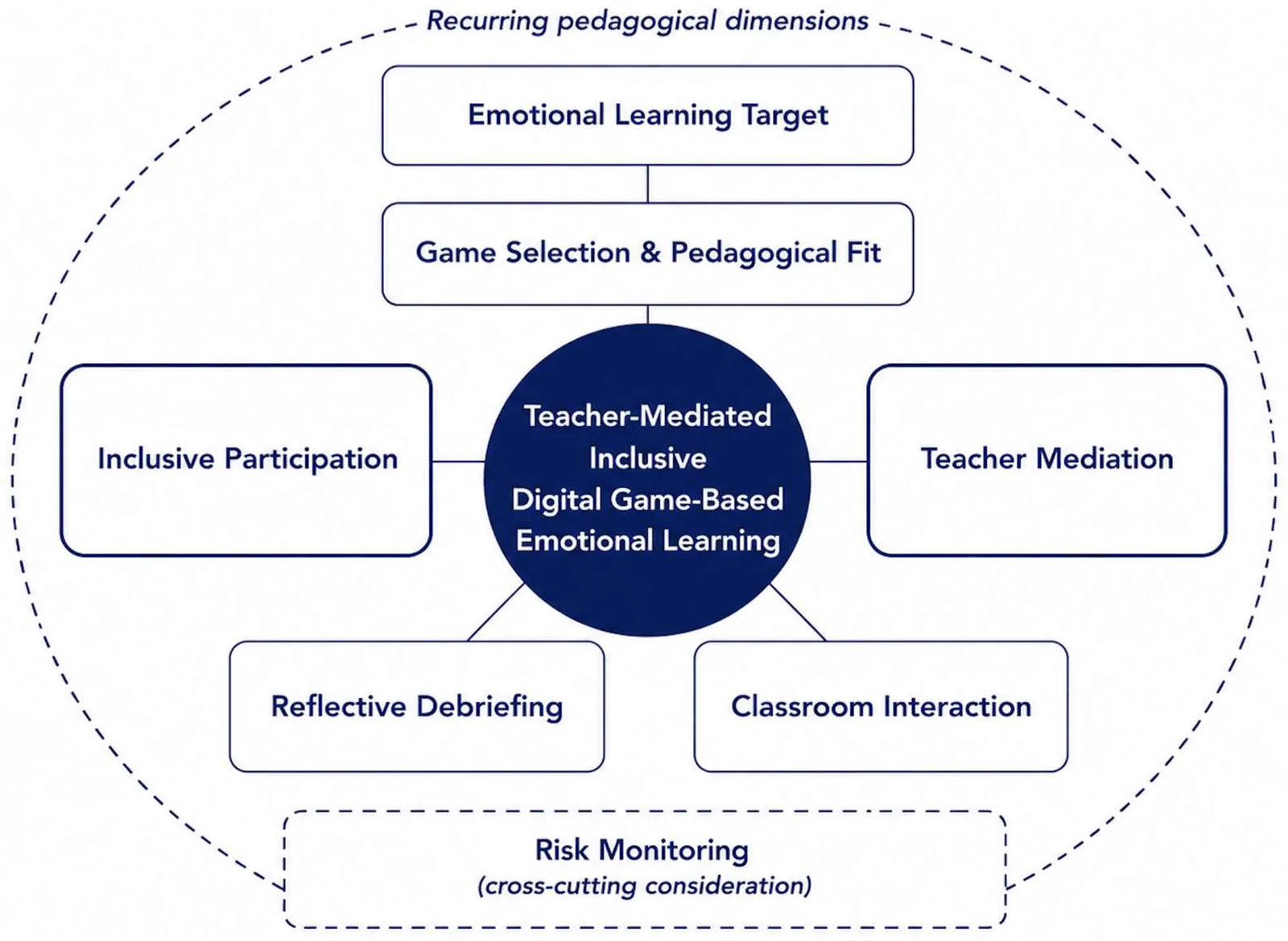
Proposed Teacher-Mediated Inclusive Digital Game-Based Emotional Learning Framework derived from the critical narrative synthesis.

Risk monitoring is positioned as a cross-cutting consideration across the framework. It concerns the identification and management of emotional-safety concerns, accessibility barriers, sensitive content, frustration, exclusion, and other implementation challenges. This dimension was derived primarily from reported limitations and implementation conditions rather than from standardized risk-monitoring procedures (Craig et al., 2015; Filella Guiu et al., 2016; Ghanat et al., 2025; Manitsa et al., 2024; Meurer et al., 2026; Paracha et al., 2023; Rihlah et al., 2026; Rončević Zubković et al., 2022; Ros-Morente et al., 2018). It is therefore presented as a responsible implementation consideration rather than as a validated intervention component. Figure 2 summarizes the seven dimensions as interrelated considerations for planning, implementing, and evaluating inclusive digital game-based emotional and social competency activities. The available evidence does not establish that all seven dimensions are required in every context or that any dimension is more influential than another.

## 4. Discussion

### 4.1. Interpretation of the Main Findings

This review synthesized 14 studies examining how digital game-based approaches support children’s emotional intelligence and social–emotional learning in educational or classroom-relevant contexts. The findings suggest that digital games should not be interpreted as inherently beneficial emotional learning tools. Rather, their contribution depends on how they are pedagogically structured, mediated, and connected to explicit emotional or social–emotional learning goals. Across the included studies, digital games were used to support emotional competence, emotion regulation, empathy, compassion, social skills, prosocial behaviour, resilience, and inclusive participation (Filella Guiu et al., 2016; Meurer et al., 2026; Ros-Morente et al., 2018; Saleme et al., 2021).

A recurring pattern across the included studies was that digital games appeared more educationally meaningful when they functioned as structured social and emotional scenarios rather than as isolated technological activities. For example, serious games and gamified activities addressing empathy, bullying prevention, and prosocial behaviour created opportunities for children to engage with social dilemmas, recognize others’ emotions, and reflect on consequences (Paracha et al., 2020, 2023; Rončević Zubković et al., 2022; Saleme et al., 2020, 2021). Similarly, gamified emotional education software was associated with emotional competence, peer conflict resolution, and well-being when embedded in structured emotional education processes (Filella Guiu et al., 2016; Ros-Morente et al., 2018). These findings indicate that digital games may be particularly relevant to emotional intelligence when they provide opportunities for emotional practice, social interpretation, and guided reflection. However, the results were not consistent across studies. Some studies reported only limited improvements, some found effects that were not clearly linked to the game itself, and others did not show clear mediation effects (Meurer et al., 2026; Rončević Zubković et al., 2022; Ros-Morente et al., 2018; Saleme et al., 2020).

### 4.2. Pedagogical and Inclusive Implications

The findings highlight that teacher or facilitator mediation may be an important condition for meaningful digital game-based emotional learning. The included studies suggest that emotional and social learning appeared more likely to be supported when adults prepare students for gameplay, structure participation, guide interaction, and connect game experiences to real-life emotional and peer-related situations. This was particularly visible in studies using computer-game supported classroom activities, emotional education software, school-based interventions and trauma-informed programmes (Chao-Fernández et al., 2020; Meurer et al., 2026; Ros-Morente et al., 2018). Hence, the role of the teacher might extend beyond technical supervision to pedagogical mediation to help students interpret gameplay in relation to emotional and social experiences. However, teacher or facilitator mediation was seldom studied as an independent variable and its specific contribution needs to be tested.

The review also shows that inclusive participation is not a natural product of the use of digital games. Inclusion depends on accessibility, differentiated participation, emotional safety, and sensitivity to learner diversity. Studies involving students with vision impairment or hearing impairment showed how accessible design and supportive implementation can enable social inclusion and emotion recognition (Ghanat et al., 2025; Manitsa et al., 2024). Together, these studies raise important considerations regarding accessibility and implementation, but their small, context-specific samples and developmental design limit broader conclusions. Research on bullying prevention, empathy, and peer interaction suggests that digital games can also contribute to safer and more reflective classroom environments when used to promote compassion, perspective-taking, and prosocial responses (Paracha et al., 2023; Rončević Zubković et al., 2022; Saleme et al., 2021).

These findings provide support for the proposed Teacher-Mediated Inclusive Digital Game-Based Emotional Learning Framework. The framework emphasizes seven interrelated dimensions: emotional learning target, game selection and pedagogical fit, teacher mediation, inclusive participation, classroom interaction, reflective debriefing, and risk monitoring. This framework may guide educators, researchers, and designers in moving beyond the question of whether digital games are effective toward the more pedagogically meaningful question of under what conditions they support emotional intelligence and social–emotional learning.

### 4.3. Risks, Boundary Conditions, and Adjacent Evidence

The review also identified important boundary conditions. Although the final synthesis focused on classroom-relevant and pedagogically structured studies, several excluded studies showed that digital games are also used in adjacent therapeutic, clinical, parent-supported, or mental health-related contexts. Studies involving autism-focused serious games, psychoeducational depression prevention, conduct problems, emotional regulation assessment, or problematic gaming were excluded from the final synthesis because they did not sufficiently align with the review’s classroom-based and teacher-mediated focus (Beaumont et al., 2021; Bohr et al., 2023; David et al., 2018, 2024; Hassan et al., 2025; Livanou et al., 2025). However, these studies remain useful for understanding the broader landscape of digital games and emotional development.

This distinction is important because educational and therapeutic uses of digital games may overlap but should not be conflated. A game designed for clinical emotion regulation or autism-centre intervention may offer valuable insights into digital emotional learning, but its implementation logic differs from teacher-mediated classroom practice. Similarly, risk-oriented studies on problematic gaming, screen exposure, or emotional dysregulation highlight the need to monitor adverse effects, but they cannot be treated as direct evidence for school-based emotional learning. Therefore, the present review positions risk monitoring as an essential component of responsible classroom use. Digital games may support emotional intelligence, but only when teachers attend to frustration, exclusion, excessive competition, social comparison, emotional discomfort, and accessibility barriers.

### 4.4. Limitations and Future Research

This review has several limitations. First, the search was limited to Web of Science and Scopus and to English-language articles and early access articles published between 2015 and 2026. This may have excluded relevant conference papers, non-English studies, national publications, and emerging educational technology work. Second, the BiBLoX-exported working dataset contained incomplete abstract metadata for some records. To reduce premature exclusion, uncertain cases were retained for full-text assessment, but metadata incompleteness remains a limitation. Third, the included studies were heterogeneous in terms of educational level, game type, research design, intervention duration, and outcome measures. For this reason, a meta-analysis was not appropriate, and a critical narrative synthesis was used instead.

A further limitation concerns the proposed seven-dimensional framework, which is an interpretive product of the critical narrative synthesis rather than a validated measurement model or intervention protocol. Because reporting of teacher mediation, reflective debriefing, accessibility, and risk monitoring was inconsistent, the framework should be viewed as a theoretically informed guide requiring prospective validation in classroom research.

Future research should examine teacher-mediated digital game-based emotional learning through classroom-based, longitudinal, and comparative designs. More studies are needed on how teachers facilitate reflection after gameplay, how inclusive participation is supported among diverse learners, and how game features such as competition, scoring, collaboration, feedback, and narrative design affect emotional safety and peer interaction. Future studies should also develop clearer assessment tools for emotional intelligence and social–emotional outcomes in digital game-based learning contexts.

## 5. Conclusions

This systematic review examined how teacher-mediated digital game-based approaches support or constrain children’s emotional intelligence and social–emotional learning in inclusive classroom-relevant contexts. The findings indicate that, under certain pedagogical and contextual conditions, digital games may provide opportunities for emotional practice, empathy, peer interaction, emotion recognition, emotion regulation, prosocial behaviour, resilience, and social inclusion. However, these benefits are not produced by digital games alone. They depend on the pedagogical conditions under which games are selected, structured, facilitated, and reflected upon.

The review identifies teacher or facilitator mediation as a recurring and potentially important condition for responsible digital game-based emotional learning. Digital games appear to be more educationally meaningful when they are connected to explicit emotional or social–emotional learning goals, embedded in structured classroom or school-based activities, and followed by guided reflection. However, the available evidence does not establish teacher mediation as an independently tested causal mechanism, and its specific contribution requires further investigation.

The findings also show that inclusive participation requires careful attention to accessibility, differentiated engagement, emotional safety, and learner diversity. Studies involving students with sensory impairments and diverse social–emotional needs demonstrate that digital games can support inclusion when participation barriers are addressed. At the same time, emotionally sensitive content, competition, frustration, exclusion, social comparison, and problematic engagement must be monitored carefully.

The Teacher-Mediated Inclusive Digital Game-Based Emotional Learning Framework proposed in this review offers a practical synthesis for educators, researchers, and designers. It emphasizes seven interrelated dimensions: emotional learning target, game selection and pedagogical fit, teacher mediation, inclusive participation, classroom interaction, reflective debriefing, and risk monitoring. Future research should further examine these dimensions through classroom-based, longitudinal, and comparative studies that investigate not only whether digital games work, but also how, for whom, and under what pedagogical conditions they support children’s emotional intelligence.

## Figures and Tables

**Table 1:** Operationalized inclusion and exclusion criteria used during title–abstract screening and full-text eligibility assessment.

Criterion Domain	Include Studies When…	Exclude Studies When…	Indicators Used During Screening
1. Type of game-based intervention	The study examines digital games, serious games, educational games, learning games, game-based learning environments, gamified learning activities, mobile games, video games, computer games, virtual reality games, augmented reality games, or educational gamification used for educational, developmental, or social–emotional purposes.	The study focuses on non-educational gaming, general media use, non-digital play, or digital technology use without a game-based or gamified component.	Terms such as digital game, serious game, educational game, game-based learning, gamified learning, video game, mobile game, VR game, AR game, gamification.
2. Emotion-related constructs	The study addresses at least one emotion-related or social–emotional construct, such as emotional intelligence, emotion recognition, emotion regulation, empathy, emotional competence, social–emotional competence, emotional development, social skills, peer interaction, cooperation, classroom climate, or emotional well-being.	The study addresses only academic performance, cognitive achievement, technical usability, or general motivation without any emotion-related or social–emotional outcome.	Terms such as emotional intelligence, empathy, emotion regulation, social skills, social–emotional learning, emotional well-being, peer interaction, classroom climate.
3. Population/learner group	The study involves children, adolescents, pupils, school students, or learners in preschool, kindergarten, primary, elementary, middle school, secondary school, lower-secondary, or K-12 contexts.	The study focuses exclusively on adults, university students, higher education learners, or mixed samples in which school-age data cannot be distinguished.	Terms such as child, adolescent, school student, preschool, elementary, primary school, middle school, secondary school, K-12.
4. Educational context	The study is situated in a school-based or pedagogically structured context, or provides transferable evidence for school-age emotional learning.	The study concerns home gaming, recreational gaming, clinical therapy, informal leisure use, or general online gaming without a pedagogical or educational context.	Terms such as school-based, classroom, educational setting, intervention, curriculum, learning activity, teacher-led, classroom implementation.
5. Teacher mediation/pedagogical structure	The study includes, or clearly implies, a teacher, educator, facilitator, or structured instructional role in selecting, organizing, facilitating, or debriefing the digital game-based activity.	The study presents gaming only as an individual, unsupervised, or purely recreational activity with no educational guidance or pedagogical structure.	Terms such as teacher, educator, facilitator, instructional design, teacher-mediated, guided activity, debriefing, classroom facilitation.
6. Methodological sufficiency	The study provides sufficient information about participants, educational context, intervention or learning activity, research design, data collection, and findings to support systematic analysis.	The study lacks basic methodological information, reports only minimal descriptive content, or does not allow the educational/game-based and emotional dimensions to be evaluated.	Presence of a clear sample, setting, intervention description, method, and findings in the abstract and/or full text.
7. Risk-only or pathology-only focus	The study may be retained if it discusses risk, frustration, exclusion, or problematic aspects together with a school-based, pedagogical, or game-based educational component relevant to emotional or social learning.	The study is excluded if it focuses solely on gaming disorder, problematic gaming, digital addiction, aggression, screen-time exposure, or psychopathology without a classroom-based or pedagogical component.	Terms such as problematic gaming, gaming disorder, aggression, addiction, screen time were used as exclusion signals unless accompanied by a clear educational intervention or classroom context.

**Table 2:** Study, participant, design, intervention, and outcome characteristics of the 14 primary studies included in the critical narrative synthesis.

Study	Country/Context	Participants	Design and Methods	Precise Evidential Contribution
Paracha et al. (2019)	Japan; two elementary-school participatory-design workshops in Oita	Two groups of 30 children; total contributor *N* = 60; ages 7–12; sex NR	Action research and participatory design using fictional inquiry, comic-boarding, role-play, classroom discussion, digital storyboarding, prototype work, and qualitative template analysis	Children generated bullying scenarios, character, interface, storyline, and coping-strategy ideas. The study reported design-related evidence concerning ethical reasoning, reflection, empathy, and children’s agency; it did not test intervention effectiveness.
Filella Guiu et al. (2016)	Spain; ten primary schools, comprising six experimental schools and four control schools	*N* = 574 fifth- and sixth-grade students: 301 boys (52.4%) and 273 girls (47.6%); mean age = 10.53 years (*SD* = 0.662). The experimental group included 351 students, and the control group included 223 students.	School-based quasi-experimental pretest–post-test study with a control group. Teachers completed 30 h of training, and outcomes were assessed using emotional competence, anxiety, classroom and playground climate, conflict records, academic performance, and student–teacher evaluation measures.	Fewer playground conflicts and selected gains in classroom climate and Spanish-language achievement. However, the non-randomized design limits causal interpretation.
Saleme et al. (2020)	Australia; three private schools in a large metropolitan city	Recruited *N* = 389; matched pre–post sample *N* = 364; Years 3–6; ages 8–12 years; 85.2% boys and 13.7% girls.	Mixed-method, one-group pretest–post-test pilot. Children completed a 45 min REMI programme combining an interactive comic book and augmented reality. Surveys were analyzed using paired-samples t-tests, and facilitator observations and participant feedback were analyzed thematically.	Overall empathy, affective empathy, and empathic behavioural intentions increased significantly, whereas cognitive empathy and social norms did not change significantly. The uncontrolled, single-session design supports only short-term preliminary evidence.
Ros-Morente et al. (2018)	Spain; primary and secondary schools	Total *N* = 1477. Primary: *N* = 574, 301 boys and 273 girls, mean age 10.53. Secondary: *N* = 903, 471 male and 432 female, mean age 12.63	School-based quasi-experimental pretest–post-test study with experimental and control groups. Students used Happy 8–12 or Happy 12–16 during one academic year. Emotional competence, state anxiety, and academic achievement were analyzed using ANOVA.	Happy 8–12 was associated with improved emotion regulation, reduced state anxiety, and higher Spanish-language achievement. Happy 12–16 was associated with improvements in emotional awareness, emotional autonomy, life competencies, and academic achievement; however, state anxiety increased and changes in total emotional competence, emotion regulation, and social competence were not significant. The non-randomized design and absence of long-term follow-up limit causal interpretation.
Rončević Zubković et al. (2022)	Ten European schools: five in Spain, three in Malta, one in the United Kingdom, and one in Ireland	School of Empathy group: *N* = 120, ages 12–14 years (*M* = 12.75, *SD* = 0.70), 61 boys and 59 girls. Control-game group: *N* = 116 (*M* = 12.65, *SD* = 0.72), 54 boys and 62 girls.	Pretest–post-test comparative study. Students played either the School of Empathy bullying-prevention game or an internet-safety control game for 4–6 h. Online surveys assessed knowledge, compassion, and game experience; structural equation modelling and moderated mediation analyses were conducted.	The proposed mediation effects were not supported. In the School of Empathy group, perceived challenge positively predicted postgame knowledge, while immersion positively predicted compassion. These relationships were not significant in the control-game group, although initial knowledge and compassion remained strong predictors of postgame outcomes.
Saleme et al. (2021)	Australia; theory-based development of a digital game for children aged 8–11 years	Stakeholders included teachers and education experts, game designers and developers, a child counsellor, and behaviour-change researchers. Four co-design sessions involved 6–10 children per session; the total number of unique children and sex distribution were NR.	Intervention-development study using the six-step Intervention Mapping Protocol. The R.E.M.I. prototype was co-designed through literature review, stakeholder consultation, Living Lab sessions, and child feedback. It included five decision-making scenarios, goal setting, feedback, and a post-game discussion.	The study produced a theory-informed digital game prototype targeting self-awareness, empathy, perspective-taking, collaboration, helping, and prosocial behaviour. It provides design, feasibility, and implementation evidence, but no effectiveness outcomes because the proposed pretest–post-test evaluation had not yet been conducted.
Paracha et al. (2020)	Japan; child-centred virtual-environment development	Multiple cohorts: conception workshop (*N* = 30, ages 7–12); narrative workshop (*N* = 30, ages 7–12); formative evaluations (>80 children across four sessions); summative evaluation (*N* = 20, ages 10–12); sex NR.	Participatory design, fictional inquiry, comic-boarding, classroom discussion, prototype exposure, questionnaires, and usability evaluation	Produced usability and design findings relating to moral, social, and emotional-learning scenarios. The study did not provide controlled evidence of competency improvement.
Craig et al. (2015)	United States and Japan; Grades 3–4	Total *N* = 497; United States *N* = 270; Japan *N* = 227; approximately ages 7–11; female(full sample) = 47.7% female(Japan) = 46.7%, female(US) = 43.0%.	Cross-cultural game-based social-skills assessment using behavioural game tasks and multivariate comparisons	Japanese participants scored higher on emotion regulation and cooperation; US participants scored higher on empathy and social initiation; impulse-control differences were not significant. This was an assessment study, not an intervention trial.
Chao-Fernández et al. (2020)	Spain; secondary education	5 males, 1 female; age range 16–18 years (mean = 17.16 years)	Small pilot with pre–post emotional and behavioural assessment during computer-game-supported music activities	Reported improvements in selected emotional-intelligence-related measures and reductions in inappropriate or disruptive behaviours. The very small, uncontrolled sample restricts causal and generalizable conclusions.
Paracha et al. (2023)	Japan; elementary-school settings in Oita and Yufuin, Kyushu	Total *N* = 80 children. Participatory Design Workshop I: *n* = 30, ages 7–12; Workshop II: *n* = 30, ages 7–12; summative evaluation: *n* = 20, ages 10–12. The groups were separate; sex distribution NR.	Four-phase qualitative action-research study involving conceptualization, participatory design, iterative development, and evaluation of the Shimpai Muyou serious game. Methods included fictional inquiry, comic-boarding, interactive theatre, digital storyboarding, classroom discussions, direct observation, video and log data, focus groups, template analysis, and log analysis.	Participatory-design and gameplay findings indicated increased bullying awareness, empathic engagement with game characters, reflection on moral dilemmas, perceived usability, and consideration of coping and conflict-resolution strategies. As an exploratory qualitative study without a control group, it provides design, usability, and implementation evidence rather than causal evidence of effectiveness.
Manitsa et al. (2024)	United Kingdom; development of an inclusive school game	Phase 1 included four adolescents with vision impairment, an eight-member youth advisory group, and six professionals; Phase 3 included five adolescents with vision impairment and professional contributors; target age 12–16; sex NR in the accessible summary	Multi-phase participatory development using interviews, focus groups, prototype work, and reflexive thematic analysis	Produced design and acceptability evidence concerning coping, self-advocacy, school participation, accessibility, and social inclusion. It did not establish effectiveness through a controlled outcome trial.
Ghanat et al. (2025)	Iran; two speech therapy centres in Arak city	*N* = 30 hard-of-hearing children aged 6–10 years (*M* = 7.86, *SD* = 1.52); 13 boys and 17 girls. Experimental group: *n* = 15; control group: *n* = 15.	Quasi-experimental pretest–post-test design with a control group and purposive sampling. The experimental group completed twelve 30 min EMOFIT sessions over five weeks, while the control group continued standard centre activities. Facial-emotion recognition and understanding were assessed, and the results were analyzed using MANOVA.	EMOFIT significantly improved facial-emotion recognition and understanding. Effects were stronger for emotion understanding (η^2^ = 0.85) than recognition (η^2^ = 0.80), with the largest gain in adaptation (η^2^ = 0.84) and the smallest in emotion naming (η^2^ = 0.57). The small, context-specific sample limits generalizability.
Rihlah et al. (2026)	Indonesia; several *Pondok Pesantren* and early-childhood education settings in Surabaya	Effectiveness sample: *N* = 81 children aged 4–6 years; 27 in the Si Robot Pintar group, 27 in the interactive-video group, and 27 in the worksheet control group. Sex distribution NR. An additional empirical instrument test involved 200 children.	Research-and-development study using the ADDIE framework, followed by a quasi-experimental pretest–post-test design with two experimental groups and one control group. Participants were selected purposively and assigned according to existing classroom placements. Outcomes included identifying and controlling emotions, cognitive abilities, and character development.	Section 3 reported significant improvements in cognitive abilities and character development, particularly emotion identification and control. The Si Robot Pintar group showed a large effect size (partial η^2^ = 0.890), compared with 0.875 for interactive videos and 0.540 for worksheets. Non-random assignment and the context-specific sample limit causal interpretation and generalizability.
Meurer et al. (2026)	United States; four Milwaukee schools	*N* = 329 students selected for survey data and 321 completed; qualitative components included 57 students participated in focus groups, 68 students participated in interviews, and 29 teachers participated in focus groups; 53% boys, 41% girls, 3% nonbinary/gender fluid/intersex, 1% other, 2% preferred not to answer (*n* = 321 surveyed); target age range 10–15 years (Grades 5–10)	Clustered crossover incomplete-block factorial randomized mixed-methods trial of the multicomponent STRYV365 programme	Peak Team was associated with improved emotional regulation and Brain Agents with improved coping in reported regression analyses. Because gameplay was embedded within a multicomponent programme, the independent contribution of the game cannot be isolated.

Note. *N* = total sample size; *n* = subgroup or phase-specific sample size; *M* = mean; *SD* = standard deviation; NR = not reported; PD = participatory design; SEL = social–emotional learning; ADDIE = Analysis, Design, Development, Implementation, and Evaluation; Sex and gender terminology follows the wording used in the original studies. Participant numbers in participatory-design, development, usability, and feasibility studies refer to study contributors and should not necessarily be interpreted as intervention-effectiveness samples.

**Table 3:** Code-to-dimension audit trail for the proposed Teacher-Mediated Inclusive Digital Game-Based Emotional Learning Framework.

Framework Dimension	Contributing Analytic Codes	Code-to-Dimension Consolidation Logic	Included Studies Contributing to the Dimension
1. Emotional learning target	Emotional awareness; emotional understanding; emotion recognition; emotion regulation; emotional competence; empathy; compassion; prosocial behaviour; social skills; peer interaction; resilience; well-being; character development; social inclusion	Codes specifying what learners were expected to recognize, regulate, understand, practise, or develop were consolidated into the intended emotional or social–emotional learning target.	Craig et al. (2015); Filella Guiu et al. (2016); Ros-Morente et al. (2018); Paracha et al. (2019, 2020, 2023); Saleme et al. (2020, 2021); Chao-Fernández et al. (2020); Rončević Zubković et al. (2022); Manitsa et al. (2024); Ghanat et al. (2025); Meurer et al. (2026); Rihlah et al. (2026).
2. Game selection and pedagogical fit	Game format; intervention purpose; scenario type; game mechanics; assessment versus instruction; developmental suitability; educational level; accessibility; alignment between the game activity and target outcome	Codes describing what the game required learners to do and whether its content, mechanics, format, and level were aligned with the intended emotional outcome were consolidated into pedagogical fit.	Craig et al. (2015); Filella Guiu et al. (2016); Ros-Morente et al. (2018); Paracha et al. (2019, 2020, 2023); Saleme et al. (2020, 2021); Chao-Fernández et al. (2020); Rončević Zubković et al. (2022); Manitsa et al. (2024); Ghanat et al. (2025); Meurer et al. (2026); Rihlah et al. (2026).
3. Teacher mediation	Preparation for gameplay; instructions; structured participation; facilitator guidance; programme integration; curriculum alignment; adult support; guided interpretation; instructional follow-up	Codes representing actions through which a teacher, facilitator, mentor, or structured programme organized and connected gameplay to educational goals were consolidated into teacher or facilitator mediation.	Filella Guiu et al. (2016); Ros-Morente et al. (2018); Paracha et al. (2019, 2020, 2023); Saleme et al. (2020, 2021); Chao-Fernández et al. (2020); Rončević Zubković et al. (2022); Manitsa et al. (2024); Ghanat et al. (2025); Meurer et al. (2026); Rihlah et al. (2026).
4. Inclusive participation	Accessibility; differentiated participation; sensory support; learner diversity; equitable access; emotional safety; participation barriers; social inclusion	Codes concerning whether learners with different sensory, developmental, social, or emotional needs could access and participate safely and meaningfully were consolidated into inclusive participation.	Craig et al. (2015); Filella Guiu et al. (2016); Paracha et al. (2023); Rončević Zubković et al. (2022); Manitsa et al. (2024); Ghanat et al. (2025); Meurer et al. (2026); Rihlah et al. (2026).
5. Classroom interaction	Peer collaboration; peer conflict; social participation; perspective-taking; compassion; bullying awareness; moral or ethical reasoning; prosocial responses; disruptive behaviour; classroom climate	Codes describing how gameplay shaped interaction among learners or provided scenarios for interpreting and responding to other people were consolidated into classroom interaction.	Craig et al. (2015); Filella Guiu et al. (2016); Paracha et al. (2019, 2020, 2023); Saleme et al. (2020, 2021); Chao-Fernández et al. (2020); Rončević Zubković et al. (2022); Manitsa et al. (2024).
6. Reflective debriefing	Guided discussion; interpretation of emotions; reflection on choices and consequences; transfer from the game to real-life situations; facilitated follow-up; mentorship; processing emotionally sensitive scenarios	Codes representing formal debriefing or functionally equivalent processes through which learners interpreted the gameplay experience and connected it to emotional or social situations were consolidated into reflective debriefing. Formal debriefing was not consistently labelled or reported across the studies.	Filella Guiu et al. (2016); Paracha et al. (2019, 2020, 2023); Saleme et al. (2020, 2021); Rončević Zubković et al. (2022); Meurer et al. (2026).
7. Risk monitoring	Emotional discomfort; frustration; exclusion; accessibility barriers; sensitive content; implementation sustainability	Codes concerning possible adverse experiences, participation barriers, methodological limitations, and implementation constraints were consolidated into a cross-cutting risk-monitoring dimension. This dimension was derived primarily from reported limitations and boundary conditions rather than from a risk-monitoring procedure tested consistently in every study.	Craig et al. (2015); Filella Guiu et al. (2016); Ros-Morente et al. (2018); Paracha et al. (2023); Rončević Zubković et al. (2022); Manitsa et al. (2024); Ghanat et al. (2025); Meurer et al. (2026); Rihlah et al. (2026).

## Data Availability

The data supporting the findings of this study are available from the corresponding author upon reasonable request. The data were derived from publicly accessible bibliographic records retrieved from Web of Science and Scopus.

## Supplementary Materials

The following supporting information can be downloaded at: https://www.mdpi.com/article/10.3390/jintelligence14080181/s1, Supplementary File S1: PRISMA 2020 checklist; Supplementary Table S1: full-text eligibility decisions.

## Appendix A. Database Search Strings

The database-specific search strings used in Web of Science and Scopus are presented in Table A1. The search strings were designed to combine three conceptual blocks: emotion-related constructs, digital game-based learning constructs, and child/school-related educational contexts. Publication year, language, and document type restrictions were applied through database refinement filters after the initial search.

**Table A1:** Database-specific search strings used in Web of Science and Scopus.

Database	Search Field	Search String
Web of Science	Topic Search TS=	TS=(((“emotional intelligence” OR “social emotional learning” OR “social-emotional learning” OR “social and emotional learning” OR “emotion regulation” OR “emotional regulation” OR “emotion recognition” OR “emotional competence” OR “social emotional competence” OR “social-emotional competence” OR “emotional development” OR empath*) AND (“digital game” OR “digital games” OR “serious game” OR “serious games” OR “educational game” OR “educational games” OR “learning game” OR “learning games” OR “game-based learning” OR “game based learning” OR “digital game-based learning” OR “digital game based learning” OR “video game” OR “video games” OR videogame OR videogames OR “computer game” OR “computer games” OR “mobile game” OR “mobile games” OR “virtual reality game” OR “virtual reality games” OR “augmented reality game” OR “augmented reality games” OR “VR game” OR “VR games” OR “AR game” OR “AR games” OR “gamified learning” OR “educational gamification” OR “gameful learning” OR (gamif* NEAR/3 learn*) OR (gamif* NEAR/3 educat*) OR (gamif* NEAR/3 classroom*) OR (gamif* NEAR/3 school*)) AND (child* OR adolescen* OR pupil* OR “school student” OR “school students” OR preschool* OR kindergarten OR “early childhood” OR “primary school” OR “primary education” OR “elementary school” OR “elementary education” OR “middle school” OR “secondary school” OR “lower secondary” OR classroom* OR school* OR “K-12” OR K12)))
Scopus	Title, Abstract, Keywords TITLE-ABS-KEY	TITLE-ABS-KEY(((“emotional intelligence” OR “social emotional learning” OR “social-emotional learning” OR “social and emotional learning” OR “emotion regulation” OR “emotional regulation” OR “emotion recognition” OR “emotional competence” OR “social emotional competence” OR “social-emotional competence” OR “emotional development” OR empath*) AND (“digital game” OR “digital games” OR “serious game” OR “serious games” OR “educational game” OR “educational games” OR “learning game” OR “learning games” OR “game-based learning” OR “game based learning” OR “digital game-based learning” OR “digital game based learning” OR “video game” OR “video games” OR videogame OR videogames OR “computer game” OR “computer games” OR “mobile game” OR “mobile games” OR “virtual reality game” OR “virtual reality games” OR “augmented reality game” OR “augmented reality games” OR “VR game” OR “VR games” OR “AR game” OR “AR games” OR “gamified learning” OR “educational gamification” OR “gameful learning” OR (gamif* W/3 learn*) OR (gamif* W/3 educat*) OR (gamif* W/3 classroom*) OR (gamif* W/3 school*)) AND (child* OR adolescen* OR pupil* OR “school student” OR “school students” OR preschool* OR kindergarten OR “early childhood” OR “primary school” OR “primary education” OR “elementary school” OR “elementary education” OR “middle school” OR “secondary school” OR “lower secondary” OR classroom* OR school* OR “K-12” OR K12)))

Note. The search strings did not include publication year, language, or document type restrictions. These limits were applied after retrieval through database filters: publication years 2015–2026, English language, and document type limited to Article and Article, Early Access. WoS and Scopus use different proximity operators; therefore, NEAR/3 was used in Web of Science and W/3 was used in Scopus.
