# Supplementary material for: Teacher-Mediated Digital Game-Based Learning and Children’s Emotional Intelligence in Inclusive Classrooms: A Systematic Review and Critical Narrative Synthesis
Source: J Intell. 2026 Aug 7;14(8):181. doi: 10.3390/jintelligence14080181 (PMC13514583; doi:10.3390/jintelligence14080181)
Supplement: Supplementary file 1 [file jintelligence-14-00181-s001.zip › Supplementary File S1 PRISMA 2020 checklist_updated.pdf]

PRISMA 2020 Checklist

Section and Topic	Item #	Checklist item	Location where item is reported
**TITLE**			
Title	1	Identify the report as a systematic review.	Title page: the manuscript title identifies the report as a systematic review.
**ABSTRACT**			
Abstract	2	See the PRISMA 2020 for Abstracts checklist.	Abstract: review rationale, databases and date limits, screening and inclusion counts, synthesis focus, main findings, and proposed framework.
**INTRODUCTION**			
Rationale	3	Describe the rationale for the review in the context of existing knowledge.	Introduction, Section 1: rationale, theoretical background, and the gap in existing evidence.
Objectives	4	Provide an explicit statement of the objective(s) or question(s) the review addresses.	Introduction, Section 1, final paragraph: review scope and the four interrelated issues addressed.
**METHODS**			
Eligibility criteria	5	Specify the inclusion and exclusion criteria for the review and how studies were grouped for the syntheses.	Methods, Section 2.4 and Table 1: inclusion and exclusion criteria; Methods, Section 2.8: grouping for the critical narrative synthesis.
Information sources	6	Specify all databases, registers, websites, organisations, reference lists and other sources searched or consulted to identify studies. Specify the date when each source was last searched or consulted	Methods, Section 2.2: Web of Science and Scopus. The date on which each database was last searched is not reported.
Search strategy	7	Present the full search strategies for all databases, registers and websites, including any filters and limits used.	Methods, Section 2.2; Appendix A, Table A: complete Web of Science and Scopus search strings. Year, language, and document-type limits are reported in the Appendix note.
Selection process	8	Specify the methods used to decide whether a study met the inclusion criteria of the review, including how many reviewers screened each record and each report retrieved, whether they worked independently, and if applicable, details of automation tools used in the process.	Methods, Sections 2.3, 2.4, and 2.6; Figure 1: BiBLoX-supported organization and manual two-stage screening. The number of screeners and whether screening was performed independently are reported in 2.6.
Data collection process	9	Specify the methods used to collect data from reports, including how many reviewers collected data from each report, whether they worked independently, any processes for obtaining or confirming data from study investigators, and if applicable, details of automation tools used in the process.	Methods, Section 2.7: structured extraction form and extracted variables; Section 2.8: two subject-matter experts collaboratively conducted descriptive and analytic coding. The number of data extractors, independent extraction procedures, and investigator contact procedures are explicitly reported in 2.7.
Data items	10a	List and define all outcomes for which data were sought. Specify whether all results that were compatible with each outcome domain in each study were sought (e.g. for all measures, time points, analyses), and if not, the methods used to decide which results to collect.	Methods, Section 2.4 and Table 1; Section 2.7: emotion-related and social-emotional outcome domains, reported constructs, instruments, directions of findings, and supporting quantitative or qualitative

PRISMA 2020 Checklist

Section and Topic	Item #	Checklist item	Location where item is reported
			evidence.
	10b	List and define all other variables for which data were sought (e.g. participant and intervention characteristics, funding sources). Describe any assumptions made about any missing or unclear information.	Methods, Section 2.7: context, setting, participants, design, sampling, intervention, comparator, implementation, facilitator role, data collection, measures, findings, attrition, and limitations. Missing information was recorded as NR and was not inferred. Funding source was not listed as an extracted study-level variable.
Study risk of bias assessment	11	Specify the methods used to assess risk of bias in the included studies, including details of the tool(s) used, how many reviewers assessed each study and whether they worked independently, and if applicable, details of automation tools used in the process.	Methods, Sections 2.8-2.9; Results, Section Methodological Quality and Limitations of the Included Evidence : no formal risk-of-bias tool was applied; a design-sensitive descriptive appraisal across six domains was used.
Effect measures	12	Specify for each outcome the effect measure(s) (e.g. risk ratio, mean difference) used in the synthesis or presentation of results.	Methods, Sections 2.7-2.8: study-reported effect estimates, test statistics, confidence intervals, significance levels, and descriptive changes were extracted where available. No common effect measure or pooled estimate was used.
Synthesis methods	13a	Describe the processes used to decide which studies were eligible for each synthesis (e.g. tabulating the study intervention characteristics and comparing against the planned groups for each synthesis (item #5)).	Methods, Sections 2.4 and 2.8; Results, Sections 3.3-3.8: studies were compared and grouped by game-based approach, outcome focus, pedagogical and inclusion conditions, risks, and framework dimensions.
	13b	Describe any methods required to prepare the data for presentation or synthesis, such as handling of missing summary statistics, or data conversions.	Methods, Sections 2.7-2.8: missing information was recorded as NR, characteristics were not inferred, and extracted quantitative and qualitative evidence was organized without statistical data conversion.
	13c	Describe any methods used to tabulate or visually display results of individual studies and syntheses.	Results, Figure 1; Tables 2-4; Figure 2. Table 2 presents study characteristics and findings, and Table 3 presents the code-to-dimension audit trail.
	13d	Describe any methods used to synthesize results and provide a rationale for the choice(s). If meta-analysis was performed, describe the model(s), method(s) to identify the presence and extent of statistical heterogeneity, and software package(s) used.	Methods, Section 2.8: rationale and procedures for the critical narrative synthesis, including iterative comparison, descriptive and analytic coding, thematic grouping, and framework development.
	13e	Describe any methods used to explore possible causes of heterogeneity among study results (e.g. subgroup analysis, meta-regression).	Methods, Section 2.8; Results, Sections Methodological Quality and Limitations of the Included Evidence and 3.7: heterogeneity was explored narratively across populations, educational levels, interventions, game formats,

Section and Topic	Item #	Checklist item	Location where item is reported
			contexts, outcomes, measures, and study designs. No subgroup analysis or meta-regression was conducted.
	13f	Describe any sensitivity analyses conducted to assess robustness of the synthesized results.	Not applicable. No sensitivity analyses were conducted or reported because no statistical synthesis or meta-analysis was performed.
Reporting bias assessment	14	Describe any methods used to assess risk of bias due to missing results in a synthesis (arising from reporting biases).	Not reported. No formal assessment of bias due to missing results or other reporting biases was conducted.
Certainty assessment	15	Describe any methods used to assess certainty (or confidence) in the body of evidence for an outcome.	Not reported. No formal certainty-of-evidence assessment, such as GRADE, was conducted.
**RESULTS**			
Study selection	16a	Describe the results of the search and selection process, from the number of records identified in the search to the number of studies included in the review, ideally using a flow diagram.	Results, Section 3.1 and Figure 1 shows identification, accessibility and retrieval, deduplication, screening, full-text assessment, exclusion, and final inclusion counts.
	16b	Cite studies that might appear to meet the inclusion criteria, but which were excluded, and explain why they were excluded.	Results, Section 3.1 and Discussion, Section 4.3: selected excluded studies are cited as adjacent evidence. A study-level list citing all 17 excluded reports is provided as Supplementary Table S2 Full Text Eligibility Decisions.
Study characteristics	17	Cite each included study and present its characteristics.	Results, Section 3.2 and Table 2: all 14 included studies are cited and their contexts, participants, designs, methods, and evidential contributions are presented.
Risk of bias in studies	18	Present assessments of risk of bias for each included study.	Methods, Section 2.9; Results, Section Methodological Quality and Limitations of the Included Evidence and Table 2: design-sensitive methodological limitations are appraised narratively. No formal per-study risk-of-bias ratings or standardized tool results are presented.
Results of individual studies	19	For all outcomes, present, for each study: (a) summary statistics for each group (where appropriate) and (b) an effect estimate and its precision (e.g. confidence/credible interval), ideally using structured tables or plots.	Results, Table 2 and Sections 3.3-3.7: study-level findings and study-reported quantitative or qualitative evidence are presented. Uniform group summary statistics and precision estimates were not available or reported for every study.
Results of syntheses	20a	For each synthesis, briefly summarise the characteristics and risk of bias among contributing studies.	Results, Section Methodological Quality and Limitations of the Included Evidence , Sections 3.3-3.8, Table 2, and Table 3:

Section and Topic	Item #	Checklist item	Location where item is reported
			contributing study characteristics, methodological limitations, thematic results, and framework contributions are summarized.
	20b	Present results of all statistical syntheses conducted. If meta-analysis was done, present for each the summary estimate and its precision (e.g. confidence/credible interval) and measures of statistical heterogeneity. If comparing groups, describe the direction of the effect.	Not applicable. No statistical synthesis or meta-analysis was conducted; therefore, no pooled effect estimate, precision measure, or statistical heterogeneity estimate was calculated. Individual-study quantitative findings are presented in Table 2 and the narrative Results sections.
	20c	Present results of all investigations of possible causes of heterogeneity among study results.	Results, Sections Methodological Quality and Limitations of the Included Evidence and 3.3–3.7. Possible sources of heterogeneity were examined narratively across study design, participant characteristics, educational context, intervention and game format, intervention duration, outcome constructs, measurement approaches, and teacher or facilitator involvement. No statistical subgroup analysis or meta-regression was conducted.
	20d	Present results of all sensitivity analyses conducted to assess the robustness of the synthesized results.	Not applicable. No formal sensitivity analyses were conducted to assess the robustness of the narrative synthesis.
Reporting biases	21	Present assessments of risk of bias due to missing results (arising from reporting biases) for each synthesis assessed.	Not reported. No formal assessment of reporting bias due to missing results was conducted.
Certainty of evidence	22	Present assessments of certainty (or confidence) in the body of evidence for each outcome assessed.	Not reported. No formal certainty-of-evidence assessment was conducted.
**DISCUSSION**			
Discussion	23a	Provide a general interpretation of the results in the context of other evidence.	Discussion, Sections 4.1-4.3: interpretation of the findings in relation to broader pedagogical, inclusive, and adjacent evidence.
	23b	Discuss any limitations of the evidence included in the review.	Results, Section Methodological Quality and Limitations of the Included Evidence ; Discussion, Section 4.4: limitations of the included evidence and the proposed framework.
	23c	Discuss any limitations of the review processes used.	Discussion, Section 4.4: limitations of the search scope, language and publication-type restrictions, BiBLoX metadata availability, heterogeneity, and narrative synthesis.
	23d	Discuss implications of the results for practice, policy, and future research.	Discussion, Sections 4.2 and 4.4; Section 5, Conclusions: implications for educational practice, design,

PRISMA 2020 Checklist

Section and Topic	Item #	Checklist item	Location where item is reported
			responsible implementation, and future research. Policy implications are not discussed separately.
**OTHER INFORMATION**			
Registration and protocol	24a	Provide registration information for the review, including register name and registration number, or state that the review was not registered.	Methods, Section 2.1: the review was not prospectively registered.
	24b	Indicate where the review protocol can be accessed, or state that a protocol was not prepared.	Methods, Section 2.1: no separate review protocol was published.
	24c	Describe and explain any amendments to information provided at registration or in the protocol.	Not applicable. The review was not registered and no separate protocol was prepared; therefore, no registration- or protocol-based amendments are reported.
Support	25	Describe sources of financial or non-financial support for the review, and the role of the funders or sponsors in the review.	Funding statement: the review received no external funding; therefore, no funder or sponsor role is reported.
Competing interests	26	Declare any competing interests of review authors.	Conflicts of Interest statement: the authors declare no conflict of interest.
Availability of data, code and other materials	27	Report which of the following are publicly available and where they can be found: template data collection forms; data extracted from included studies; data used for all analyses; analytic code; any other materials used in the review.	Data Availability Statement: supporting data are available from the corresponding author upon reasonable request and were derived from Web of Science and Scopus records. Supplementary Materials: PRISMA 2020 checklist. No public repository, extraction form, analytic code, or complete screening dataset is identified.

From: Page MJ, McKenzie JE, Bossuyt PM, Boutron I, Hoffmann TC, Mulrow CD, et al. The PRISMA 2020 statement: an updated guideline for reporting systematic reviews. BMJ 2021;372:n71. doi: 10.1136/bmj.n71

For more information, visit: http://www.prisma-statement.org/
