# Supplementary material for: Teacher-Mediated Digital Game-Based Learning and Children’s Emotional Intelligence in Inclusive Classrooms: A Systematic Review and Critical Narrative Synthesis
Source: J Intell. 2026 Aug 7;14(8):181. doi: 10.3390/jintelligence14080181 (PMC13514583; doi:10.3390/jintelligence14080181)
Supplement: Supplementary file 1 [file jintelligence-14-00181-s001.zip › Supplementary_Table_S1_Full_Text_Eligibility_Decisions.pdf]

Supplementary Table S1. Full-Text Eligibility Decisions

No	Study	Decision	Eligibility basis / reason
1	Grossard et al. (2017a), *Serious games to teach social interactions and emotions to individuals with autism spectrum disorders (ASD)*	Excluded	Relevant to serious games, emotions, and ASD; however, the classroom-based and teacher-mediated implementation was weak or unclear.
2	Beaumont et al. (2021), *Randomized Controlled Trial of a Video Gaming-Based Social Skills Program for Children on the Autism Spectrum*	Excluded	Video-gaming-based social skills programme for children with autism, but primarily parent-supported or therapeutic rather than classroom-based or teacher-mediated.
3	Paracha et al. (2019), *Co-design with Children: Using Participatory Design for Design Thinking and Social and Emotional Learning*	Included	Met the criteria for child participation, pedagogically structured design, SEL relevance, empathy/ethical reasoning, and facilitator-supported learning.
4	David et al. (2018), *REThink online video game… The Feeling Better resources game*	Excluded	Relevant to emotion understanding and regulation, but framed primarily as a therapeutic video-game environment rather than a classroom-based pedagogical intervention.
5	Muravevskaia and Gardner-McCune (2023), *Designing a Virtual Reality Empathy Game Framework…*	Excluded	Relevant to empathy and VR game design for children, but design/framework-oriented with weak school/classroom implementation evidence.
6	Filella Guiu et al. (2016), *Happy 8-12*	Included	Met the criteria for school-age learners, emotional education software, peer conflict resolution, and emotional competence development.
7	Saleme et al. (2020), *A Gamified Approach to Promoting Empathy in Children*	Included	Met the criteria for child-focused gamification, empathy promotion, and social–emotional learning relevance.
8	Ros-Morente et al. (2018), *Analysis of the Effects of Two Gamified Emotional Education Softwares…*	Included	Met the criteria for gamified emotional education software, children/adolescents, emotional variables, and well-being outcomes.
9	Rončević Zubković et al. (2022), *Bullying Prevention Serious Game…*	Included	Met the criteria for serious game use, bullying prevention, compassion, peer-related socio-emotional outcomes, and educational relevance.
10	Saleme et al. (2021), *Digital Game Intervention to Promote Socio-Emotional Skills and Prosocial Behavior in Children*	Included	Met the criteria for digital game intervention, child sample, socio-emotional skills, prosocial behavior, and pedagogical design relevance.
11	Paracha et al. (2020), *Virtual Environment on Moral, Social, and Emotional Learning*	Included	Met the criteria for serious/virtual game-based learning, moral/social/emotional learning, child-oriented design, and pedagogical structuring.
12	Craig et al. (2015), *Zoo U: A Game-Based Social Skills Assessment*	Included	Included as assessment-focused evidence; met the criteria for school-age children, game-based social skills assessment, and socio-emotional competence relevance.

No	Study	Decision	Eligibility basis / reason
13	Chao-Fernández et al. (2020), *Computer-Game-Based Music…*	Included	Met the criteria for secondary education, computer-game-based emotional training, disruptive behavior modification, and educational context.
14	Bohr et al. (2023), *Psychoeducational Serious Game SPARX…*	Excluded	Serious game and youth mental-health relevance, but primarily psychoeducational/mental-health oriented rather than classroom-mediated SEL.
15	Grossard et al. (2017b), *JEMImE Project / Emotional Expressions for Children with ASD*	Excluded	Relevant to emotion recognition and ASD, but framed as reeducation/design in a specialized developmental or clinical context, with weak teacher/classroom mediation.
16	Hernandez-Lara et al. (2023), *Emotion4Down*	Excluded	Serious video game for emotional awareness and intellectual disability; school/classroom and teacher-mediated context was insufficiently aligned with the review focus.
17	David et al. (2024), *Gamified Assessment of Emotion Regulation Abilities in Youth*	Excluded	Relevant to emotion regulation assessment, but primarily validation/assessment-oriented and not a classroom-based digital game intervention.
18	Black et al. (2025), *Gaming in the Intervention and Support Process…*	Excluded	Relevant to gaming and diverse needs, but closer to therapy or facilitated support than classroom-based teacher mediation.
19	Paracha et al. (2023), *Shimpai Muyou… Serious Game & School-Based Intervention*	Included	Met the criteria for serious game use, school-based intervention, child well-being, bullying/suicide-prevention relevance, and social–emotional risk awareness.
20	Çar et al. (2023), *8-Week Educational Game Training… Emotional Intelligence and Coping Strategies*	Excluded	Relevant to emotional intelligence and coping, but the game component was not clearly digital, computer-based, mobile, or video-game based.
21	Álvarez-Muñoz et al. (2025), *Responsible Use of Video Games in Primary Education…*	Excluded	Relevant to responsible video-game use and socio-emotional e-competence, but focused mainly on family supervision and use patterns rather than classroom-based game intervention.
22	Manitsa et al. (2024), *Vi-Connect… Social Inclusion at School of Students with Vision Impairment*	Included	Met the criteria for educational game development, school inclusion, students with vision impairment, and inclusive participation.
23	Grunewald et al. (2025), *Gamified Cognitive Training on Adolescent Mental Health*	Excluded	Relevant to gamified training and adolescent mental health, but weakly classroom-mediated and closer to mental-health training than school-based emotional learning.
24	Livanou et al. (2025), *ReGoal… Serious Mobile Game for Young People with Conduct Problems*	Excluded	Serious mobile game for conduct problems, but clinical/support-oriented with weak classroom or teacher-mediated implementation.
25	Gómez-León (2024), *Effectiveness of Video Games in the Emotional Regulation of Students*	Excluded	Systematic review and meta-analysis rather than a primary empirical study; retained only for background and contextual discussion.

No	Study	Decision	Eligibility basis / reason
26	Hassan et al. (2025), *Zirkus Empathico 2.0… Children with ASD*	Excluded	Strong serious mobile game intervention for ASD and social-emotional development, but conducted in autism-center/intervention settings rather than classroom-based teacher mediation.
27	Ghanat et al. (2025), *Computer Game for Recognizing and Understanding Facial Emotions…*	Included	Met the criteria for computer game use, emotion recognition, children with hearing impairment, and inclusive social–emotional learning relevance.
28	Nagpal et al. (2026), *FriendZi… Game-Based Curriculum for Elementary Students' Social-Emotional Wellbeing*	Excluded	Does not meet the criteria for a digital game study
29	Rihlah et al. (2026), *AI-Based Edugame "Si Robot Pintar"… Early Childhood Children*	Included	Included as peripheral SEL/character-development evidence; met the criteria for early childhood education, edugame use, and character/social development relevance.
30	Meurer et al. (2026), *STRYV365 Trauma-Informed School Program*	Included	Met the criteria for school-based programme, game-based learning, mentorship, resilience, emotional intelligence, and trauma-informed emotional support.
31	Novianto et al. (2026), *Numeracy Activities Integrated with Pancasila Values in Mobile Games*	Excluded	Mobile-game and elementary context present, but the main focus was numeracy and values education; emotional intelligence/SEL and teacher mediation were indirect or weak.
